# On the nature of evolutionary explanations: a critical appraisal of Walter Bock’s approach with a new revised proposal

**DOI:** 10.1007/s40656-023-00601-7

**Published:** 2024-01-08

**Authors:** Marcelo Domingos de Santis

**Affiliations:** 1 Departamento de Entomologia, Museu Nacional, UFRJ, Rio de Janeiro, RJ Brazil; 2 Museum Koenig Bonn, Leibniz-Institut zur Analyse des Bioaffiliationersitatswandels, Adenauerallee 127, 53113 Bonn, Germany

**Keywords:** Deductive-nomological model, Narrative explanation, Historical explanation, Evolutionary explanation, Evolutionary biology

## Abstract

Walter Bock was committed to developing a framework for evolutionary biology. Bock repeatedly discussed how evolutionary explanations should be considered within the realm of Hempel’s deductive-nomological model of scientific explanations. Explanation in evolution would then consist of functional and evolutionary explanations, and within the latter, an explanation can be of nomological-deductive and historical narrative explanations. Thus, a complete evolutionary explanation should include, first, a deductive functional analysis, and then proceed through nomological and historical evolutionary explanations. However, I will argue that his views on the deductive proprieties of functional analysis and the deductive-nomological parts of evolution fail because of the nature of evolution, which contains a historical element that the logic of deduction and Hempel’s converting law model do not compass. Conversely, Bock’s historical approach gives a critical consideration of the historical narrative element of evolutionary explanation, which is fundamental to the methodology of the historical nature of evolutionary theory. Herein, I will expand and discuss a modern view of evolutionary explanations of traits that includes the currentacknowledgement of the differences between experimental and the historical sciences, including the token and type event dichotomy, that mutually illuminate each other in order to give us a well confirmed and coherent hypothesis for evolutionary explanations. Within this framework, I will argue that the duality of evolutionary explanations is related to two components of character evolution: origin, with its evolutionary pathways along with the history, and maintenance, the function (mainly a current function) for the character being selected.

*In science, explanation is everything.*

Bock (1991, p. 9).

## Introduction

There was a time when biologists felt that they should stand up in defense of an autonomous biology, or, more precisely, an autonomous evolutionary biology. Popper was among those philosophers of science that claimed that the theory of natural selection is tautological (Popper, 1972) and that “…Darwinism is not a testable scientific theory, but a metaphysical research programme…” (Popper, 1974, p. 134), thus, Popper put the evolutionary theory in great jeopardy. In addition, the essentially historical nature of theories of phylogenetic history, as a result of natural selection, was an additional blow to evolutionary biology, as Popper, alongside the philosophers of its time, argued that scientific theories cannot be historical hypotheses. As these hypotheses incorporates a causal narrative structure, it does not fit the laws of nature demanded of a scientific theory along Popper’s views. Hence, the whole discipline of phylogenetics would not count as a genuine science according to Popper; in the words of Stamos (2007, p. 366): “In genuine science, for Popper, including historical sciences, the ‘‘science’’ is only in the use of laws of nature. No testing of laws, or no use of laws for explanation or prediction, and no science.” These views were in accordance with the idea of physics as the “fundamental science”, on the basis which all other scientific concepts should be defined, being, indeed, the paradigm science. Thus, as evolutionary theory does not conform to the pattern he found in physics, it would not be a testable scientific theory. This was true for the logical positivists, the logical empiricists, and for most philosophers from the first half of the twentieth century, most prominently in the two of the most influential philosophers of science of that century: Karl R. Popper, as discussed, and Carl G. Hempel.

The fight back from the evolutionary biologists, mainly from the 1960s onward, was conducted by some noteworthy scientists such as Ernst Mayr (1969, 1982, 1996), George Gaylord Simpson (1963a, b) and Francisco José Ayala (1968, 1995), that were in the vanguard of biologists whom seriously explored and scrutinized philosophical themes in their works. The overall agreement that these scientists reached is that evolutionary biology is a scientific research program not despite being historical in nature, it is scientific because of its historical particularities. The philosophers of science, with their narrow view of what a scientific discipline is, should change their approach in order to include evolution as a scientific endeavor, and not the other way around, i.e., all that the evolutionary biologists were doing, with all its success, is not scientific and are less valuable than what physicists were doing. Furthermore, they argued that biology is not reducible to physics (nor chemistry), because its objects (organisms) cannot be reduced to objects of the inanimate world. This is due to the emergent proprieties of organisms, in Mayr’s (1982, p. 63) words, these being: “Systems [that] almost always have the peculiarity that the characteristics of the whole cannot (not even in theory) be deduced from the most complete knowledge of the components, taken separately or in other partial combinations.” Finally, they argued that in relation to the universal laws, as used by physics, are relatively unimportance for biology, or are even nonexistent. Let us see, for instance, Simpson (1980, p. 122) on laws in Zoology: “There was a phase when many zoologists… believed that most phylogenetic problems might be solved by a set of rather simple rules or so-called “laws” … However, those… rules are not laws but only statistical generalizations which cannot be assumed to hold good in any particular instance. It has indeed been found that some proposed “rules” have more exceptions than examples.” Today, it is widely recognized the scientific status of evolutionary biology among the philosophers of science and biology (e.g., Brandon, 1990; Hull, 1974; Sober, 2000; Uller & Laland, 2019; Delisle, 2021).

Nevertheless, some of those evolutionary biologists took another strategy and instead of showing that biology is autonomous and independent of other sciences (mainly physics) and a particular view of a philosopher, insisted on placing biology and evolutionary biology within the tenets of Popper’s and Hempel’s philosophy. Popper exerted the most influence upon evolutionary biologists for the defense of evolutionary biology as a science; they tried to construct evolution within the views of Popper’s “demarcation problem,” the quest for what distinguishes science from nonscience and pseudoscience by his criteria of falsifiability, even though the problems with Popper’s solution are well known (for instance, the Duhem-Quine problem). Thus, many scientists working with evolution argued, sometimes vigorously, that the structure of evolutionary biology is in the form of the hypothetico-deductive method (closely associated with the philosophy of Karl Popper). Sometimes, even the authors that argued for the autonomy of biology, later, tried to include evolutionary theory as a hypothetical-deductive theory, for instance, Ayala (2009) and more distinctly Ghiselin (1969) in his book, “*The triumph of the Darwinian Method*”. Especially interesting for the porpoises of this paper, is the use of Popper’s philosophy of science to settle foundational issues in phylogenetic systematics. Initially, it was intended to settle the “systematics at war” period against the rival’s school of classification but was later used in the debate of those who defend cladistic parsimony methods and those who defend maximum likelihood methods (for a detailed discussion see, Hull, 1988 and Helfenbein & DeSalle, 2005). However, as it was acknowledged since the 1970s, the historical science of phylogenetics does not proceed through deductive logic, nor is a hypothetical-deductive science, and finally, it does not contain any laws as devised by Popper (e.g., Hull, 1983; Sober, 1988; Rieppel, 2003; Vogt, 2008; Santis, 2020a and references therein). This rather uncontroversial conclusion was reached for biology in general and in contemporary philosophy of science, where Popper's influence definitely seems to be fading (Godfrey-Smith, 2016). Even with this criticism, why did natural scientists recur by Popper’s philosophy to defend their ideas? One possible reason was explored by Godfrey-Smith (2016, p. 104), arguing that: “Popper offers a rather heroic view of the scientific character, featuring an appealing combination of creativity and hard-headedness.” Then he adds that (Godfrey-Smith, 2016, p. 104): “It is no surprise that they [scientists] prefer this picture to the one often (though inaccurately) associated with Hempel and Carnap – the scientist as a sort of logic-driven pattern recognition machine.” However, perhaps surprisingly, there were some evolutionists, and cladists, that tried to defend their idea of evolution as a genuine science by using the logicist and logical empiricist Carl Hempel. Although it was used by the cladist Arnaud Kluge to defend his approach for cladistics (e.g., 1999, 2009; for a detailed criticism seeSantis, 2020a), it was better developed and more clearly exposed by the, sadly recently deceased (Schmitt & Buckeridge, 2022), Walter Joseph Bock (1934–2022) throughout his career in numerous publications. Bock, who was also interested in the autonomy of biology (e.g., Bock, 2017), repeatedly claimed that there are two types of equally contrasting forms of explanation occurring in evolutionary explanations (Bock, 1999, p. 46): “These dual sets are the dichotomy of Nomological-Deductive Explanations (N-D E) versus Historical-Narrative Explanations (H-N E) and the dichotomy of Functional Explanations (F E) versus Evolutionary Explanations (E E).” Adding further that (Bock, 1999, p. 46): “All functional explanations are nomological-deductive, and all historical-narrative explanations are evolutionary. But there is an overlap between these two sets because evolutionary explanations can be either nomological-deductive or historical narrative.” Finally, these are within the realm of science because (Bock, 2000, p. 35): “Both N-D E and H-N E are scientific under the criterion of demarcation for scientific explanations advocated by Popper in that both are available for testing by falsification against empirical, objective observations.” Contrarily to earlier authors, Bock was not interested only in the scientific confirmation of evolutionary biology (that for him was also Popperian), but also in its scientific explanation. For that, he used the philosophy of Ernest Nagel and mainly Carl Hempel.

Finally, while critically evaluating the methodological and epistemological status of the study of adaptation, Olson and Arroyo-Santos (2015) developed what they called “five myths of hypothetico-deductive evolutionary biology”. They argued that deduction and the hypothetico-deductive method are myths, alongside with myths of the Popperian philosophy of science and how evolutionary biologists use Popper’s falsification. Olson and Arroyo-Santos (2015) were occupied with the *scientific confirmation* of hypotheses, while this paper will deal with the *scientific explanation* of evolutionary hypothesis *sensu* Bock. Hence, herein I will add a further myth: that the deductive-nomological model of scientific explanation, or the covering-low model, of Hempel is not adequate to accommodate evolutionary explanations.

In order to espouse these issues, I will proceed as follows. Firstly, I will provide, synthetically, the ideas of Walter Bock on functional explanations and evolutionary biology that are nomological-deductive in nature according to Bock’s view, in addition to scrutinize his use of Hempel’s philosophy, whether explicitly or implicitly stated. Next, I will show how, similarly to Popper, this connection of evolution to the deductive-nomological model of scientific explanation does not hold in evolutionary explanations, neither from the functional explanation nor the deductive-nomological models. I will explain the historical nature of the structure of evolutionary theory, and I will argue that even if Bock’s procedure fails, his notion of functional and evolutionary explanations being required for full explanations in biology, is correct and it should be carefully considered by any scientist trying to give an explanation of a trait in an organism in evolutionary studies. I will further argue that a more fruitful view, that is in accordance with the nature of the evolutionary theory, is one that includes both experimental and the historical sciences that are present in the methodology and epistemic of evolutionary studies. Additionally, the notion of a type and token event will be valuable to further characterize evolutionary explanations. Bock’s development of historical-narrative explanations will be important to complemental the historical aspect of evolutionary studies. I will provide a comprehensive picture of evolutionary explanations of traits, and for an exemplification of the arguments discussed, will include the scientific dispute over the best explanation for the origin and evolution of insect wings.

## Bock on evolutionary explanations

Walter Bock got his Ph.D., from Harvard University in 1959 and his thesis advisor was none other than Ernst Mayr (Schmitt & Buckeridge, 2022), one of the main architects of the “Moderns Synthesis” of evolution; they remained close for the rest of their careers and lives. Bock was one of the main exponents of the traditional view called “evolutionary taxonomist” or gradist school of classification (e.g., Bock, 1979). In addition, by introducing Popper’s views on the science of classification and phylogenetic inference, Bock (1973) initiated the discussion on the philosophical ground on which science would be better along the Popperian philosophy of science. Thus, Bock (1973) argued that classical evolutionary classification should prevail as a classification system over phenetics and cladistics because of its consistency with Popper’s ideas. This paper was the open venue that witnessed the strike back from cladists that claimed that, contrarily to Bock’s assertion, cladistics was instead the only research program that was in accordance with Popper’s principles (see, e.g., Hull, 1988; Rieppel, 2008; Santis, 2020b). Another discussion brought up by Bock was the issue of explanation of adaptation in biology (Bock & von Wahlert, 1965). These studies led Bock to develop a keen interest in the nature of explanations in morphology. For Bock, an explanation of a trait is a dichotomy between functional biology versus evolutionary biology (very closely related to the proximal versus ultimate causation of Mayr, 1961). Functional analysis is ahistorical according to Bock, being independent of evolutionary explanations. What is more, he considered that (Bock, 1988, p. 207): “Functional explanations in biology use nomological-deductive analysis as their basic method of study, that is, the formulation of hypotheses and testing predictions deduced from these hypotheses against empirical observations the basic deductive approach in science.” Bock further adds that in any biological system, functional explanations can only be complete as long as it associated with their historical origins; evolutionary explanations fulfil this desideratum by giving hypotheses about the origin of an evolutionary change of a particular trait. There are some methodological differences within an evolutionary explanation; thus, Bock tells us that an additional dichotomy must be done to accurately determine which kind of explanation we are dealing with. If it is of nomological-deductive or historical-narrative nature. Similarly to Mayr, Bock constructed his dual notion of explanation in evolution as a matter of “how” and “why”. In Bock’s (1999, p. 49) words: “Functional explanations cover “how” explanations in morphology—namely how attributes of organisms work or operate and how these attributes develop ontogenetically. Evolutionary explanations are the “why” explanations in morphology—namely why attributes of organisms came into being originally and have modified (= evolved) over historical time (e.g., longer than one generation).” In the next section, I will focus on scrutinizing the structure of the evolutionary explanation as devised by Bock.

### The dualistic form of evolutionary explanations

Bock (2007b, p. 279) states quite clearly his motivations behind developing a deductive-nomological approach to the structure of evolutionary biology: “If evolutionary theory is exclusively historical, the major difficulty is how to consider it to be scientific, realizing that most philosophers of science regard historical analyses and the inductive method as lying outside the realm of science. The major thrust of my paper was to demonstrate the connection between nomological evolutionary theory and historical evolutionary theory in order to bring the latter within the realm of science.” Thus, all his constructions were a necessity to put evolution within the “realm of science” even if by this, as it will be argued, some “transformations” on what constitutes a correct deductive-nomological in evolution is had to be done. Thus, Bock tells us that deductive nomological explanations are universal statements that are temporally-spatially unrestricted (e.g., 1991, 1999, 2004, 2007a); as it is thought to occur in physics (but see Cartwright, 1983). However, he adds that universally should be restricted, for biology, to (Bock, 1999, p. 47) “the “surface”—the upper part of the crust—of the earth.” Bock’s idea of limiting the universality of laws seems at odds with the general usage of the term by Popper and Hempel, and I will further explain some of these disparities afterwards. Later, Bock further adds, rightfully, that due to the nature of a D-N explanation in evolutionary biology, one must accept, almost as logical necessity, that (Bock, 1999, p. 48) “laws, or law-like statements, exist in biology.” Falsification was the way to test these D-N explanations, if it does not agree with independent empirical observations, it means that the particular N-D explanation is invalid, either because one of the laws used is invalid, or because one set of initial conditions was mistaken. Hence, Bock accepts the desideratum of the existence of laws in evolution and then goes on to espouse what exactly is D-N within the structure of evolutionary biology.

At first, Bock (1999) vaguely ascribed that evolutionary N-D explanations include the causal theory of evolution. But in later publications, Bock (2004) clearly stated which of the theories that compose the theory of evolution presents a N-D scheme. Taking the famous argument developed by Mayr (1982) that Darwin’s theory of evolution constitutes five separate but interrelated theories, Bock (2004, pp. 50–51) then explain which of these theories are deductive-nomological. Thus, (1) evolution as such (Bock, 2004, p. 51) “the theory that states that all populations of organisms are changing over time, with the minimum time period being one generation”; (2) gradualism (Bock, 2004, p. 51) “the idea that evolutionary change takes place in steps of the magnitude seen between parents and offspring and never in large sudden saltations or jumps”; (3) multiplication of species (Bock, 2004, p. 51) “that happen when there is a “splitting of phylogenetic lineages as well as transformational change within a lineage.”; and (4) the theory of natural selection, are all deductive-nomological. Of the five theories of evolution, only one, theoretical statements about the history of organisms, like common descent, are not deductive-nomological in nature; thus (Bock, 2004, p. 51) the “most aspects of evolutionary theory are not historical, but nomological-deductive”.

Evolutionary biology presents a large historical component, and Bock acknowledged this in his development of historical-narrative explanations. These explanations are designed to provide scientific explanations to (Bock, 2000, p. 36) “descriptive and explanatory phylogenies, biological classifications which are derived from explanatory phylogenies, historical biogeography, etc.” The role of this type of narrative explanation is to (Bock, 2000, p. 35) “… provide an understanding of the existing attributes of a particular set of objects at a definite point in time; these explanations depend on the past history of these objects…” Furthermore, historical-narrative explanations must be based on pertinent fundamental N-D explanations. Bock (2000, p. 41) connects his deductive and non-deductive views of evolutionary explanations as follows: “…H-D Es must be founded on well-tested N-D Es with the final testing being against objective empirical observations. It is simply not sufficient to use vaguely formulated N-D Es as the basis of H-N Es; rather one must test thoroughly the supporting N-D Es before formulating H-N Es in any science. Hence any H-N evolutionary explanation is absolutely dependent on well-tested and corroborated N-D Es, both functional and evolutionary.” Being so different from the D-N explanation scheme, Bock considers that the historical subjects of historical-narrative explanations are particulars, not universals, and are temporally-spatially restricted (this sounds much similar to Bock’s demands for universals in biology); hence, no laws of nature are needed. Bock (1988, 1991, 1999, 2000, 2004) emphasizes the importance of chronological order of events and changes to explain the history of a particular trait, being unique. This uniqueness is due to the form of the organism and not their functions, as this functional equivalence (aerodynamics) between the flight in mammals and insects, is explained by convergence and not by common descent. Moreover, although Bock (2004, 2010) strongly suggests that his historical-narrative explanations are outside of the realm of philosophers such Popper (1959) and Hempel (1965), because these explanations are not deductive, Bock (1999, p. 49) also considers that “Both N-D E and H-N E are scientific under the criterion of demarcation for scientific explanations advocated by Popper in that both are available for testing by falsification against empirical, objective observations.” Finally, one is now able to answer what constitutes a “complete exaplanation” *sensu* Bock (1988, p. 207): “…[b]y complete explanations, I mean that there are no aspects of the phenomenon which have been left unexplained.” These aspects, within evolutionary biology, are twofold, evolutionary explanations (with its historical-narrative explanations and Nomological-Deductive Explanations) with the proper prior functional explanations (with its Nomological-Deductive Explanations). In other words, in accordance with Bock, a complete evolutionary explanation should include, first, a deductive functional analysis, and then nomological and historical evolutionary theories.

### Problems with Bock’s evolutionary explanations

In order to know what exactly Bock means by a functional explanation, we must go back to one of his famous early papers, co-authored with Gerd von Wahlert, namely, *Adaptation and the Form-Function Complex* (Bock & Wahlert, 1965). In this paper, which established the meaning of some terms that would be used by Bock throughout his career, the authors redefined some widely utilized concepts like form, function and adaptation. In their definition of function, they were clear in differentiating it from another concept, the one of biological role. For them, function means only something that “… is used in the sense of the physical and chemical properties of the feature…” (Bock & Wahlert, 1965, pp. 274–275), adding that functions “…may be described and treated in the same way as a typical physical and chemical law (e.g., Nagel, 1961, p. 406)” (Bock & Wahlert, 1965, p. 275). Correctly in my view, Bock later emphasized that these functional explanations (Bock, 2000, p, 34) “…cover “how” questions in biology—namely how known attributes of organisms work or operate and how these attributes develop ontogenetically”, that is very close to Mayr (1982) notion of functional explanations in biology. For instance, Bock (2007a, p. 97) argued that explanations involving “physical-chemical properties” and “ontogenetic development” would be functional explanations. This “ontogenetic development” is what one can find in most of the evolutionary literature that deals with the mechanism of a character, for instance as it happens with the mechanistic explanations found in Evolutionary Developmental Biology, or just as Evo-Devo (Kaiser, 2021). It is possible to explain the phenotypic diversity of organisms (through its growth, differentiation, and morphogenesis) by considering the causal roles of gene expression and developmental pathways (Hall, 1999; Minelli, 2009). On the other hand, Bock and Wahlert (1965, p. 278) defined biological role “…as the action or the use of the faculty by the organism in the course of its life history.” Or as they added (Bock & Wahlert, 1965, p. 278): “…the observation of the organism living naturally in its environment.” Thus, this is the current definition most biologists and philosophers of biology mean when they are referring to functions, and functional analysis, in organisms. Accordingly, Bock even explicitly argued against the use of functional explanations “in the sense of functional explanations in philosophy”; using as an instance the philosopher of science Nagel (1961)—showing his mixed thoughts on what constitutes the structure of functional explanations. Conversely, Bock wrote elsewhere (e.g., Bock, 1999, 2007a) that all functional explanations, or, as most philosophers and biologists of today would say, mechanistic explanations, are of D-N type. However, functional explanations (*sensu* Bock) meaning how structures operate in biology, i.e., mechanical explanations, do not need to be considered within a nomological-deductive structure, see, for instance, that many of the most impressive mechanicist explanations in Evo-Devo do not need to be framed, or to follow, any aspect of the D-N structure (see, for instance, Moczek, 2020 and Wagner, 2014, or, for a philosophical perspective, Kaiser, 2021). On the other hand, functional explanations, a biological role for Bock, are legitimate as used largely in all biology and philosophy (see for instance Brandon, 1990; Ayala, 2016; Neander, 1991; Ruse, 2003; Gardner, 2009; Garson, 2019), as it gives evolutionary biology its epistemic robustness by showing how functional equivalences occur thoroughly in the tree of life. Although Bock did not give enough emphasis on how important this procedure is for evolutionary explanations, I will later argue its importance and include it as one of the positive contributions of Bock to current evolutionary theory. As a straightforward example, take the adaptations of Arctic and Antarctic mammals and birds to the challenges of polar life. The animals can regulate their body temperature by growing winter plumage (birds – except penguins) and coat of fur (mammals), or by relying on a layer of blubber to prevent heat (penguins). Each of these strategies has the function of protecting from the cold, so they are functionally equivalent (same design), even if morphologically different. This example shows teleology as a legitimate way of looking at evolutionary characters, as will be further explored later—a move that Bock did not follow because he thought of teleology as misguided by considering it only as a form of anthropomorphism and vitalism.

Before going any further, it is worthwhile to mention that the primacy of functional explanations in relation to all other explanations fails because of a lack of a methodology to discern if a function happens (morphological similarity) due to a common ancestor or only due to common environmental demands. Thus, one must distinguish between homologies (synapomorphies—shared, derived characters) and homoplasies (convergences), and the methodologies for such analysis were developed and are well-known since the last century (Hennig, 1966), that is, it requires a phylogenetic hypothesis in order to distinguish homology from homoplasy. However, as Bock was not committed to this research program, it led him to this kind of difficulty. Later, I will show that there is no direct primacy, but a reciprocal illumination between functions and the research of the phylogenetic history of traits.

The second problem with Bock’s evolutionary explanations is the demand that deductive-nomological explanations are necessary in some fields of evolutionary biology if evolution is to be considered within the realm of science. First, there are some well-known counter-examples in the nomological-deductive model, making it a fatally flawed model (see, e.g., Salmon, 1989) and causation is a way to avoid these errors and misconceptions. One main, and controversial, criticism of Hempel’s theory is the explanation/prediction symmetry, the so-called “symmetry thesis”. According to Hempel, there is, essentially, no difference between explanation and prediction. To predict something, we put together an argument and try to show that it is to be expected, though we don’t know for sure yet whether it is going to happen. When we explain something, we know that it has happened already, and we show that it could have been predicted, using an argument containing a law. Still, in the counterexample of the asymmetry thesis, evolution enables us to provide well-justified and informative explanations, without predictions. Scriven (1959) who strongly attacked this thesis by citing evolutionary biology and asserting that it furnishes explanations (of what has evolved) but not predictions (of what will evolve), made this argument. Or, to put in other words, explaining in evolutionary biology (as well as in other areas) is to look for a cause. Thus, the historical element of the evolutionary theory is a counter-example against the same D-N model that Bock is advocating for the evolutionary theory. In the same line, Bock argues for the existence of laws, or law-like statements, in biology. This is one of the most vexing problems arising in this context is the characterization of law-sentences, i.e., the problem of distinguishing between lawful and accidental generalizations (Salmon, 1989). One of the main properties of laws, herein restricted to Bock’s use that is based on Popperian and Hempelian view that was, on its way, firmly constructed within the realm of Chemistry and Physics, is that it must use unrestricted universals, i.e., hold throughout the universe (strict regularities). Thus, it is not restricted in scope; it refers to objects anywhere in the universe at any time in its history—past, present, or future. Another author attempted to give other types of definitions: laws might be those generalizations that are used to make predictions, are invariable, function in explanations, and are integrated into the best systematization of the facts (Pfeifer, 2006). While a particular view of these matters is the notion of stability in general theories, as developed by Woodward (2003). But, as Salmon (1989) argued, the problem of characterizing law-statements is one that has not gone away. One major problem with the formulation made by Hempel’s (1965) model is that his deductive logical structure of explanation captures effectively the Newtonian method of physical inquiry, for instance, using Newton’s second law and the law of universal gravitation to deduce the speed at impact from the height of the fall. This very method has shaped the sciences in the modern era (Hon & Rakover, 2001), thereby bringing severe limitations in scope and applicability outside the physical science. Philosophers of biology also discussed at large the matter of laws, and some of them consider a lack of laws in the biological sciences (e.g., Beatty, 1995), despite the fact that others used a limited version (e.g., Brandon, 1997), that affirms that we have just contingent regularities in biology (see Hamilton, 2007 for further discussion). Recapitulating the point made earlier about Bock’s restriction on universal statements to the “crust” of the earth, this is a temporal-spatial restriction (similar to the historical-narrative-explanations) that is, as shown above, at odds with what is a law in the general usage of the term by Popper and Hempel, thus explaining some of the disparities and contradictions found in his defense of this model in evolutionary biology^1^. In what follows, I will now clarify the historical narrative scheme of evolutionary explanations as developed by Bock.

### Virtues with Bock’s historical-narrative explanations

Bock (2007a) argued that the views of Popper on what a genuine science leaves the evolutionary theory outside his demarcation rule. Because there are many historical aspects within evolutionary biology, and Popper could only focus on this side, evolutionary theory would not be scientific. In line with this, Bock (2000, p. 35) considered that: “… objects explained by a H-N E are singulars, not universals, and have definite spatial-temporal positions. H-N Es are stated on a nondeductive basis with the hope of reaching the most reasonable and probable explanation for the objects studied.” This is certainly true, even for the experimental aspect of evolution, leading Bock to turn the historical aspect of evolution into a scientific endeavor. Hence, as shown earlier, Bock argued that historical-narrative explanations are scientific under the criterion of demarcation developed by Popper because they “are available to tests by falsification” (Bock, 2000, p. 35). This is a clear contradiction with Bock’s (2010) own writing that affirmed that these historical hypotheses do not depend on laws (historical laws); thus how could these hypotheses be tested within a Popperian framework if they do not present laws? Stamos (2007) argued that this is not possible within Popper’s philosophy and that his approach excluded biology as Popperian science. Although Bock recognized the particularities of historical-narrative explanations, he restricted its usefulness and denied its scientific independence from the nomological-deductive structure (with it, its laws) by stating that: “What makes H-NEs scientific … [is that] H-NEs must be based on pertinent N-DEs… If this requirement is not followed for any particular H-NE, than that explanation is not scientific.” (Bock, 2004, p. 54). But now, it is widely acknowledged that if the philosophy of science as constructed for Popper is not broad enough to include historical sciences, considering that today it is widely considered as an independent scientific methodology, and evolutionary biology, that is too bad for Popper; because, as Hull (1983, p. 178) once wrote: “All truth does not reside in the writings of Sir Karl Popper. A particular thesis about science could be important and true even if Popper never mentioned it. Conversely, even if Popper held a particular view, it still could be trivial or mistaken.” The nomological-deductive model of scientific explanation of Hempel (1965) will not be going to help us here as well. Hempel maintained that history (including the historical narratives) provides us only with “explanations sketches”, as a result of the lawless structure of history (evolutionary biology included); it could not give an explanation as it falls to have a logical deduction between the laws and the initial conditions. Thus, Hempel was fully aware of the difficulty of fitting historiographic explanations into the D-N model. Furthermore, D-N model can neither account for the compelling character of many historiographic explanations nor for the central role played by explanation in the confirmation of historical hypotheses (Cleland, 2009). The failure of the deductive-nomological model of explanation in certain features of biological explanation was explored already in the 1960s by Goudge’s (1962) *The Ascent of Life* in which he argued that narrative explanations, as they belong to the evolutionary theory, aim to explain singular events that cannot be treated in term of laws of nature. As Hull further developed into these arguments (1992, p. 77): “In historical explanations an event is not explained by subsuming it under generalization. Instead, it is explained by integrating it into an organized whole.” In the same line, Richards (1992, p. 50) affirmed that: “In history, biology, and the natural sciences, explanation must demonstrate, not what *might be* causes of certain events, but what *are* the causes.” (italics in the original). Finally, narrative explanation and understanding are a type of causal explanation of past events by making descriptions and hypotheses of historical entities as they persist through time (Hull, 1992). Evolution is historical, mainly but not only, due to its inherently nature: traits do not start from scratch, but always refashions preexisting forms (Beatty & Desjardins, 2009). This is a consequence of the view of evolution as tinkering, a well-known discussion made by Jacob (1977, p. 1164): “Evolution does not produce novelties from scratch. It works on what already exists, either transforming a system to give it new functions or combining several systems to produce a more elaborate one.” Thus, as evolutionary outcomes are dependent on ancestral starting points, the recorded evolution of all life reflects the nature of the historical process that is full of contingency (Gould, 1989, 2002), and unique pathways. So, after cleaning this issue, let us see more closely the importance of historical narratives for evolutionary biology.

“[H]istorical-narrative explanations are most important in any science having a partial historical aspect, such as biology and geology. In biology, this includes the full explanation of attributes of organisms which require a historical evolutionary basis.” (Bock, 2010, pp. 71–72), this quote from Bock clearly espouses the importance of historical narratives in the historical aspect of evolutionary biology. For the sake of clarity, the classic and well know definition of what are historical narratives was given by Gould (1989, p. 283): “Historical explanations take the form of narrative: E, the phenomenon to be explained, arose because D came before, preceded by C, B, and A. If any of these earlier stages had not occurred or had transpired in a different way, then E would not exist (or would be present in a substantially altered form, E’, requiring a different explanation).” Recently, the topic of narrative explanation in the sciences has been increasingly fleshed out by philosophers (e.g., Currie, 2013; Beatty, 2016; Reydon, 2023; Kranke, 2022). And so, Currie (2013), summarizes narrative explanations: “(1) account for some particular explanandum in terms of some causal sequence; (2) target a central subject; (3) may or may not appeal explicitly to laws or generalizations; (4) are paradigmatically, but not exclusively, historical.” Later, Currie (2013) contrasted between a ‘simple’ narrative (an event is explained by a general model, and minimal causal factors are referenced) and a ‘complex narrative’ (an event that no appeal to a general model in explanation is made; rather a unique, detailed causal sequence is employed)—snowball earth being the case for the simple case while sauropod gigantism is for the complex one. Evolutionary explanations, thus, are a complex causal narrative explanation. Additionally, Beatty (2016) draws attention to the role of contingency in historical explanations. He argues that narrative explanations account for contingent outcomes and distinguishes between contingency upon prior events and contingency per se. Thus unpredictability, which characterizes contingency, is worth it when it is not known the next steps in a historical narrative.

But this topic is not without some controversies. One of the most quoted problems with this approach is that “[h]istorical narratives can only rarely (if at all) be tested by experiment.” (Mayr, 1982, p. 521, see also Smith, 2016 and Olson & Arroyo-Santos, 2015). Indeed, it is very difficult, and at times almost impossible to test these historical narratives. Before going further, we can define testability as some hypotheses that make evidence claims (predictions) about something (directly or indirectly observed) that can be checked by observation (Sober, 1999). However, as is the case of evolutionary biology, and mainly Paleontology, some narratives are causal events more distal in time than others, so it is much more difficult to make a decision about the test evidence available. Sober (1999) raised the point that testability is a changing matter because as our understanding of the empirical world raises, untestable problems may not remain so. To make a concrete example, Turner (2016) in earlier works had predicted that we would not be able to determine the colors of the dinosaurs. However, when scientists (Vinther et al., 2008) were able to study the microstructure of fossil feathers from dinosaurs’ melanosomes, they could tell us about its coloration, more precisely, that filaments from the tail of *Sinosauropteryx* Ji and Ji, 1996 have some dark-colored stripes with reddish-brown tones. This example makes it clear that the epistemology of the historical sciences, as well as the experimental sciences, advances in their methodology and gain access to information previously unable to be obtained. But we still are at a great disadvantage to gaining access to and testing whole traits and environments. Thus, while most evolutionary narratives are currently untestable, but testable in principle, we should do two things: not give up this epistemic bet against historical sciences and be aware of the enhancement of information by new methods (in this case, new fossil findings and/or new technology). However, we should be equally aware of the tremendous dilatation of time and degradation of information that all life on Earth passed by so that lots of narratives cannot be tested in this familiar fashion. Thus, one should be careful to give all the possible test evidence for some hypotheses (in contrast to a competing one). Additionally, by removing, as previously argued, the overly restricted view that Bock conceived, that all functional explanations must be D-N structured, some very useful uses of functional explanation can be pursued, such as (Bock, 1988, p. 212) “… predicting or explaining functional properties from a knowledge of morphological form plus the form-function correlations…”. Bock (1988, p. 214), further argues that: “I cannot think of a more practical and powerful way of discovering new morphological features and principles than through theoretical attempts to explain form from function.” Thus, this constitutes a fundamental and complementary explanation for morphological analyses in evolutionary studies. These uses and importance, related to the adaptation hypothesis, will be further discussed herein.

## A modern view on evolutionary explanations

Bock was a scientist of his time; a time dominated by a philosophy of science that was sorely based on physics. Today, the philosophers of biology are freed from the shackles and the narrow vision of philosophers such as Popper and Hempel to tell scientists what a good, or a genuine science is. Conversely, many biologists still rely on those philosophers to tell them on what grounds a good or bad scientific endeavour is. For instance, this occurs for some scientists working with Biogeography (e.g., Crisp et al., 2011), phylogenetic inference (e.g., Lienau & DeSalle, 2010), Ecology (Murray, 2001) and evolutionary studies (Richter & Wirkner, 2014); highlighting the necessity to discuss these issues with the philosophically directed biologists. In relation to the topic of explanations in biology, we can find a plurality of explanations that can give us a causal explanation in various fields within biology (Brigandt, 2013; Braillard & Malaterre, 2015). One can find a causal-mechanistic fashion (see Weber, 2005) exemplification of this approach in the transmission of signals along nerve fibers), mathematical models for some experimental aspects of evolutionary biology (e.g., population genetics, quantitative genetics) and ecology, and function teleologic analysis in the explanation of traits (phylogenetic maintenance) and the historical narrative (phylogenetic origin) for the biological explanations that are historical in nature. Before elucidating these issues, I believe that Bock’s main contribution to the topic of evolutionary explanations is threefold: (1) his commitment to the view that the explanation of evolutionary transformation of traits, which will have importance to the phylogenetic practice, is complex with some layers that mutually illuminate each other; (2) his views on the importance of evolution as a historical science and (3) his emphasis on the value of functional analysis (the biological role) to complement evolutionary explanations.

I argue that instead of viewing evolutionary theory as a dichotomy between a functional and a deductive-nomological side, it would be much more illuminating to appreciate a view that encompasses the differences in the methodology and epistemic between experimental and the historical sciences. Recently some evolutionary biologists have emphatically called attention to the peculiar epistemic propriety of evolutionary theory, which lies at the borderlands between historical and experimental science (Pigliucci & Kaplan, 2006; Pigliucci, 2013; see Losos (2007) for a different but complemental view on this topic). Thus, while functional (mechanicist) analysis, as discussed by Bock, is clearly experimental in nature, phylogeny and the history of traits are within the realm of historical science. Before going any further, it is worthwhile to distingue between a token and a type event. To put it simply, types are general kinds of things, and tokens are particular instances of those same kinds. In other words, the difference is that “A token event is unique and unrepeatable; a type event may have zero, one or many instances” (Sober, 1988, p. 78). The type–token distinction as applied in the historical sciences is fundamentally important; as it is interested in inferring common cause tokens, while experimental sciences are distinctly interested in inferring common cause types^2^ (see Currie, 2018; Tucker, 2004, 2014 and Cleland, 2002 for further discussion). Additionally, experimental sciences conduct controlled and repeatable experiments that are temporally independent, and their methodological acceptance and rejection of these hypotheses depends upon the success or failure of predictions; historical sciences are about particular past events and are judged by the correctness of their causal historical explanations, and most importantly, are temporally dependent of earlier stages to our current event to be explained (Cleland, 2011; see Santis, 2021, mainly the Table 1, for further distinctions).

The duality of evolutionary explanations is related to two components of character evolution: origin and maintenance^3^. Character origin, and its transformation along history, is a topic covered by the branch of science known as Phylogenetic Systematics; this research program seeks to propose classifications (reflecting the evolutionary process) with a more objective method for the elaboration of a hypothesis of the evolutionary history of groups (ancestral-descendant relation) through a differentiated analysis of the characteristics (homologies) of a set of species including an ancestral (hypothetical) and all its descendants (e.g., Hennig, 1966; Wiley & Libermann, 2011). Character origin is then the result of a phylogenetic tree, in the form of an inference to discern between a character being homolog or homoplasious, in other words, to distingue traits that present separate causes (convergences), from those that result from preserving information from common causes (homologies). Here one can investigate the mechanicist origin of a character, which can work as a complementary explanation for a particular trait by considering its developmental pathways. For instance, a convergent trait would present different (nonhomologous) underlying generators (developmental or genetic), while a homolog would present the same (homologous) underlying generators (Gould, 2002; Wake et al., 2011).

Returning to a previous example, flight in mammals and insects is due to unique inheritance from an exclusive common ancestor to each one of these taxa, this being a token event, while the ability to fly in those taxa is due to functional equivalence (aerodynamics), a clear type event. Additionally, we may call these token events within the realm of the historical sciences, while the type events are within the realm of the experimental sciences. For the objectives of phylogeny, only the common causes (homologies) are a valuable information signal. Complementarily, maintenance is the reason, or better, the function (mainly a current function) for the character being selected. Thus, when we investigate the evolutionary maintenance of a given trait, we usually prioritize the functional factors, natural selection (or sexual selection, for instance), as the most significant factors in evolutionary research, but not the only one. We might start with the question: ‘Does this trait have a function?’ If it does not, the phylogenetic result will assist in identifying situations in which a hypothetical character is simply a structural consequence of organismal architecture, development, and allometry.

I argue for the unique character of biology, and especially evolutionary biology: that functional analysis, or teleology explanations (Bock’s biological role), is a licit and legitime way to give evolutionary explanations (Santis, 2020c); this is done through matters of maintenance and evolutionary biology and systematics should follow this path if this discipline is going to have any explanatory depth (*sensu* Garson, 2019). Hence, when we give a functional explanation of a particular trait, we are giving extremely compact causal explanations for why those traits exist; these being from the same type as emphatically discussed by Bock, i.e., of explaining functional properties derived from morphological knowledge. Functions are teleological because they are forward-directed, however without being metaphysically directed (Ayala, 2016; Ruse, 2003; Santis, 2020c). Thus, a theory of function^4^, with the aim to give a teleological-causal explanation, should take the causal-explanatory role of functions, and in this way, we can give a causal explanation for the existence of traits (Garson, 2019). It is widely recognized that both mechanistic and functional explanations are understood as causal explanations (Ayala, 2016). Lombrozo and Gwynne (2014), for example, suggest that functional explanations differ from mechanistic explanations because: “…mechanistic explanations invoke proximate causal processes directly, functional explanations do so indirectly” and “functional explanations are to some extent mechanism-independent, and they have a distinct developmental and cognitive profile” (Lombrozo & Wilkenfeld, 2019).

To complement an appropriate functional analysis, one will still depend on a well-founded hypothesis of trait origin, and a phylogenetic hypothesis can give this information; without it, we should not be able to know if a character is a homology or homoplasy and if it is a derivate or primitive that are essential information for evolutionary explanations. Thus, we have a methodological framework that is temporally directed. First, we have to be able to provide a well-confirmed phylogenetic tree (trait origin) and then we can pursue the question regarding its function, it can be because of adaptation, but can be also due to other selective regimes (stochastic or constraints). The power of a phylogenetic tree, then, lies, in addition to deriving us with a hypothesis of homology, in the ability to confirm or disconfirm hypotheses of character evolution (Brooks & McLennan, 2002), more precisely, its origin. For example, a proposal that the evolution of character x was influenced by character y is deemed incorrect if x evolved before y. Coddington (1988) provides a nice example to illustrate that point. It was thought that the orb web evolved from a primitive cob web as an adaptation for catching flies more effectively. However, a phylogenetic tree (Coddington, 1986) indicates that orb webs were ancestral to cob webs, hence, the phylogenetic analysis argues against that first interpretation, and with that, our history and narrative explanation of the evolutionary transformation of that trait. Even if a functional analysis shows that, indeed, orb webs are more efficient than cob webs at catching flies, a possible interpretation is that orb webs were an exadaptation that later became an adaptation for catching flies^5^. Of course, there is no methodological magic bullet that solves all the problems of explanations of trait evolution, but the framework discussed herein is appropriate for a subset of the evolution of characters. As Ghiselin (1997) argued, evolutionary biologists—systematists included—must go beyond a phylogenetic hypothesis and must pursue a legitimate causal explanation for any trait in question. Thus, phylogenetic trees, as abstract resumes of many evolutionary events (effects), it is imperative that one must pursue the evolutionary causes for these events in a way to search for mechanisms, processes, and functions, using every biological evidence as possible to support these results. In contrast, the question of the maintenance of a character is a question about functions, because the functional characterization of the trait says why that trait has caused it to be selected (Griffiths, 1992). Thus, it should be clear by now that the acquisition of traits and functions are separate processes, because “A species may acquire a trait which has no function at all, perhaps due to its genetic linkage with an advantageous trait, and this can acquire functions when the selective environment changes.” (Griffiths, 1992, p. 124). Consequently, the construction of phylogenetic tree represents the only first step (mainly by the origin issues), not the ultimate explanation of character evolution. In conclusion, “Phylogenies are not the end of the story, merely the end of the beginning.” (Brooks & McLennan, 2002, p. 22), however, they are essential for a comprehensive evolutionary study, because evolution is a temporal process that produces lineages with a past, present, and (indeterminate) future (Brooks & McLennan, 2002). To sum up, let us take the following quote from Minelli (1993, pp. 15–16): “Evolution does not simply mean splitting lineages (cladogenesis, speciation). It also means adaptation and constraints. When deciding which state is primitive and which is advanced between, say, the absence or presence of wings, we cannot content ourselves with pattern analysis of presences and absences in a character matrix. We also need to know something about the functional value of the wing, when present, and the possible adaptive significance of its absence.” Finally, systematics, it must be emphasized, is important because all living things are the product of history, and we can understand little about the diversity of organisms without knowledge of their history—the phylogenetic knowledge provided by systematics, thus “[e]volutionary explanation depends upon systematics” (Lewontin, 2002, p. 3).

Functions, because they are the actions of phenotypic components, should be followed by some experimental measurement in the environment for the confirmation of their proper role. This is very important because information about why characters are distributed the way they are on a cladogram is obtained by these interrelations between form and function (Lauder, 1990). Hence, after we obtain a well-supported phylogenetic tree, with the aid of functional analysis, we can able to (Lauder, 1990, p. 321): “[U]nderstand a mechanical system and the causal factors involved in its construction…” Thus, functional information is a major tool for a causal explanation of Why-questions of the presence (maintenance) of characters. This inference concludes that if we give up functional analysis, a large number of inferential hypotheses, e.g., adaptation, will be left aside as well. This happens because adaptation represents one relevant way of the expression of functional explanation in matching form with function (Gans, 1988). As Garson (2019) argued, when we give functions to traits, we give a causal explanation for why those traits exist. Functional analysis can further aid us in providing information about the interaction between an organism (characters) and its environment (host use, predation). We need to know the actual environment and interaction of a taxon in order to give a concrete functional explanation. The information from the historical narrative of the past origin of a trait constitutes thus one of the central activities of narrative reasoning in evolutionary biology in reconstructing an evolutionary pathway through the ordering of certain events that ended with a certain result. Smith (2016) described that a historical explanation of a trait evolution must be embedded in a coherent narrative explanation that requires a chronology and a causal ordering of the events, which are parts of an integrated whole. Following this argument, the fact that organisms, from the present and the past, possess environmental interactions like abiotic climatic factors, biotic environmental factors or organismal features, the evolutionary succession of environmental factors that characterize the history of an evolving lineage must be taken into consideration (Larson & Losos, 1996). All these aspects discussed pertain to the topic of evolutionary scenarios, which are (Wake, 1992, p. 257) “…proposals of general patterns of evolution, including sequences of change.” Finally, and most importantly, these scenarios must be testable hypotheses, even if indirectly testable (Gans, 1989).

### Evolutionary explanation: the origin and evolution of wings in insects

In order to capture the dual nature of evolutionary explanations present in the earlier section, in what follows I present the current hypothesis that explains the origin and functional evolution of wings in insects. I intend to show that this explanation captures the character of explanations as found in texts of practicing evolutionists.

The origin of insect wings has been difficult to resolve and is one of the more contentious topics in evolutionary biology (Engel et al., 2013). The function of wings is well understood (Grimaldi & Engel, 2005), and the flight itself is highly beneficial and adaptative for insects because it enables them to disperse across physical barriers, to be in safe places away from ground-dwelling predators, to provide access food sources that others cannot reach and, finally, it can make finding a mate a much easier task (Ross, 2017). Thus, a well-confirmed functional hypothesis is clearly available. However, stating their origin and homology (token events) is more problematic to state. One main reason for this is that the common ancestor of wing insects, or Pterygota, is thought to be nearly 400 million years (Misof et al., 2014), so any species living today is separated from this ancestral taxon by this huge amount of time, constituting a venue for processes that can destroy past pieces of evidence, and thus jeopardize many historical inferences. In the last years, however new evidence and theories have been erected that shed new light on this topic. To be able to explain the complex and difficult topic of insects’ origin it is necessary to collect the most relevant evidence concerning the phylogenetic origin of wings, namely the clade Pterygota. Engel et al. (2013) argued that one of the fundamental issues, to begin with, is to give phylogenetic information on the presence of wings in insects. As it constitutes a synapomorphic trait, with a single evolutionary origin (Beutel et al., 2017; Misof et al., 2014), one can expect the same genetic and embryological architecture to be present in all extant members of Pterygota. In addition, the phylogenetic hypothesis of extant Hexapoda (Beutel et al., 2017; Misof et al., 2014) shows us that the sister-group of Pterygota, Archaeognatha (silverfish), and the earliest extant winged insects, the Ephemeroptera (mayflies), will give us the best place of investigations and possible insights of the earlier evolution of wings. The paleontological evidence, on the other hand, shows us that potential evidence for the earlier evolution of insect wings can be extracted from the extinct lineage Palaeodictyoptera (Grimaldi & Engel, 2005). The importance of palaeodictyopterids is that they were certainly among the early diverging lineages of Pterygota (Grimaldi & Engel, 2005) and that they had prothoracic paranotal lobes, complete with venation resembling that of the flight wings (Prokop et al., 2017). Hence, all further research must be cognizant with the overall consequences of their phylogeny (character origin and transformation).

Several hypotheses to explain the origin of wings have been put forward since the last century. Among them, we had, until recently, two principal competing hypotheses: the pleural origin hypothesis in which the wings would be the thoracic serial homologues of the abdominal gills, and notal origin hypothesis in which the wings are a planar extension of the back of the exoskeleton (Engel et al., 2013). These hypotheses have been brought closer together by the consideration of a hybrid hypothesis: with both pleural and gill scenarios, in which the wing evolved from ancestral lateral extensions of the thoracic pleural, in addition to thoracic gills (Niwa et al., 2010). This dual origin hypothesis view has gained support from growing evolutionary developmental evidence, i.e., Evo-Devo (Clark-Hachtel & Tomoyasu, 2016). This hypothesis uses the combination of molecular and developmental approaches to investigate questions related to the origin of insect wings, thus constituting a mechanists explanation based on Evo-Devo. Note that here, in this mechanical explanation, we are within the realm of the experimental sciences. Niwa et al. (2010) found that both the tergal margin and pleural branches, the stylus of Archaeognatha (bristletails) and abdominal gill of Ephemeroptera (mayflies), share a part of the wing gene network of wing development with the highly derived winged insect *Drosophila*. Thus, the fusion of these two structures facilitated the evolution of the insect wing (the dual origin of insect wings). Later, Clark-Hachtel and Tomoyasu (2016, p. 82, my emphasis) expanded this hypothesis and wrote that: “…tergal expansion has provided a *genetic mechanism* responsible for a large and flat wing blade structure, while pleural plates (potentially with exite-like branches) have provided a complex articulation mechanism along with muscle attachment.” Finally, the dual origin of insect wings was further confirmed by the paleontological evidence. Prokop et al. (2017) reported a case in which three pairs of nymphal wing pads in Carboniferous species of the extinct insect order Palaeodictyoptera possessed a well-developed articulary sclerites alongside a broad fusion of the wing pads to the scutellum. This represents the first direct paleontological evidence for the dual model for insect wing origins. Hence, collectively, phylogenetic, morphological, paleontological, developmental, and genetic evidence led the scientists to conclude that wings are due to dual origin, including both gills and pleurites elements. The alternative hypotheses on the origin of wings state that wings are, instead, derived only from modified gills or only from pleura, had their tests evidence not confirmed by all those aforementioned lines of evidence, hence it was considered as lacking confirmatory instances (missing common cause token, or traces of a “smoking guns” *sensu* Cleland, 2002). Thus, by establishing the causal mechanism of wing formatting (by morphological – both on extinct and extant taxa, molecular and evo-devo evidence) it was possible to formulate test evidence to compare the alternate hypothesis of wing origin and reach a conclusion for the best explanation for the wing formation.

Finally, the narrative explanation, that provided the evolutionary scenario for the origin of wings in insects was given by Engel et al. (2013). To assess the origin of wings in insects one needs to outline hypothesized chains of events that led to the acquisition of that trait (Wake, 1992). Ancestral pterygote insects, thus probably (Engel et al., 2013, p. 292) “…lived in a seemingly barren world… in which plant life was never far from a shoreline and the climate was generally warm with a moderately high O_2_ level. Arborescence had not yet developed, and flight may have been a significant aid to reach nutritious sporangia at the apices of branches in early plants and/or for dispersal.” Considering that the most accepted hypothesis of wing evolution is of dual origin, both paranotal and gill scenarios are plausible to have happened in the evolutionary history of insects. Thus, if we consider wings originated as paranotal extensions, then (Engel et al., 2013, p. 292) “…gliding may have been the initial stage in developing flight.”. Alternatively, if the gill scenario is considered, then “…gill-like exites or gill covers expanded to catch surface breezes and carry the newly emerged adult insect along, which then evolved into structures capable of powered flight” (Grimaldi & Engel, 2005, p. 159). Clearly, there are some open issues within this narrative explanation, however, some advances proceeded as we enhance our knowledge of the deep past as inferred from fossils and minerals from that epoch (Grimaldi & Engel, 2005).

I hope to have shown how the experimental and the historical sciences, in the token and type event dichotomy, mutually illuminate each other in order to give us a well-confirmed and coherent hypothesis for the evolutionary explanation for the origin and evolution of a trait, in this case, the wings in insects. For that, it was discussed the methodological usefulness of phylogenetic systematics, the comparative morphology of paleontological data, the functional analysis of the current use of the wings, and the molecular and developmental studies that led the scientists to give weight to the evidence to a particular hypothesis (dual origin hypothesis). The inherent difficulty to proposing a strongly supported hypothesis of the evolution of a trait will never be gone, however; as these are dual historical and experimental hypotheses are complex, demanding a row of disciplines to act together to give an overall picture that can give us a reliable and supported hypothesis. Historical hypotheses are still more challenging as their evidence, the morphology and the environment of the organisms can change so much as a result of the passage of time, that the hypothesis can be undetermined in some cases. Even with these difficulties, the pursuit of explanation of traits is at the core of evolutionary biology, and the fact that evolutionary processes are not easily testable, and not easily confirmed should not be an epistemological excuse to give up to research it altogether.

## Footnotes

1 Contrarily to most evolutionary biologists, Ernst Mayr included (e.g., 1982, pp. 505–510), Bock’s asserted that of the five theories of evolution of Darwin, as exposed earlier, four of them are deductive-nomological. Although these theories can be formulated as nomological-deductive structured, it is not enlightening to pursue this logical path for the problems already discussed. Chiefly, most of Bock’s ideas can be considered as limited by his over-reliance on the D-N model.

2 Fruitful and wealthy discussions of these matters exist; being deeply rooted in the uniqueness of events as used by historical scientists. These discussions are beyond the scope of this work, but the type-token dichotomy, as defined and explained in the text, is very useful in the present context.

3 Reydon (2023) proposes a different but complemental view on this duality: for him, evolutionary explanations should compass a processual and regularity-based explanation, and a historical how-possibly explanation. Raydon’s use of how-possibly explanations, as “… *a historical description of factors that made the occurrence of the process possible in the first place*.” (Reydon, 2023, p. 181 – italics in the original), is very close to the historical narrative explanation as exposed herein. On the other hand, Reydon probably does not consider phylogenetic information much relevant, as this is not discussed at all in his paper; hence not considering the valuable components of character evolution of origin and maintenance as given by the phylogenetic hypothesis.

4 The concept of biological role of Bock and von Wahlert (1965, pp. 278–279) as the link between the form-function complexes and the environment of an organism, can be considered broad enough to include the word function as the umbrella term used herein and discussed before. This concept acts exactly as the causal-explanatory role of functions, although differing in terminology both, as argued here, do not to be constructed within a D-N structure.

5 Exadaptation is a concept defended and created by Gould and Vrba (1982). They argued that the historical genesis of a trait in contrast to its current utility. They considered trait origin, that was unrelated to their current function as very important; so much that they argued that such characters that “evolved for other usages (or for no function at all), and later “coopted” for their current role, be called exaptations.” (Gould & Vrba, 1982, p. 6). Gould and Vrba (1982) agree that various adaptations that exist today change their function along time, in other words, are a secondary adaptation of some early adaptations that had different functions; but they also considered traits that had no functions at all (nonaptations), as a phenotypic pool for natural selection to act in new adaptations.
